# Perceived Stress and Life Stressors in Adults with and without Fibromyalgia

**DOI:** 10.3390/biomedicines12061233

**Published:** 2024-06-01

**Authors:** Ha M. Nguyen, Barbara J. Cherry, Laura Zettel-Watson

**Affiliations:** 1 Department of Psychology, California State University, Fullerton, CA 92831, USA; 01nguyenh@gmail.com (H.M.N.); lzettel-watson@fullerton.edu (L.Z.-W.); 2 Aging Studies Program, California State University, Fullerton, CA 92831, USA

**Keywords:** stress, chronic pain, fibromyalgia, depression, middle-aged, older adults

## Abstract

Chronic medical conditions (i.e., chronic widespread pain) may contribute to accelerated/accentuated aging, such that middle-aged individuals with comorbidities may actually show increased declines in physical, cognitive, and mental health compared to normal aging adults. We examined perceived stress, life stressors, and depression in adults with and without fibromyalgia, a chronic pain condition. Ninety-four participants (52% with fibromyalgia, 78% female) aged 50 to 93 were administered the Perceived Stress Scale, Social Readjustment Rating Scale, and Beck Depression Inventory. Hierarchical regression analyses were conducted: the predictor variables were age, gender, fibromyalgia status, depression, and fibromyalgia–depression interaction. The interaction term significantly predicted perceived stress, but not life stressors. Depression significantly predicted stress for Social Readjustment Rating Scale measures after controlling for covariates. Significant associations were found between perceived stress and life stressors in all participants. In addition, those with fibromyalgia were significantly more likely to report higher levels of stress above standardized scores on both the Perceived Stress Scale and the Social Readjustment Rating Scale. Finally, depressive symptoms played a more significant role than fibromyalgia status in predicting life stressors. Conclusions: These findings emphasize the importance of assessing different types of stress and stressors in individuals with chronic widespread pain and/or depression in mid-life and beyond to better treat individuals with these conditions.

## 1. Introduction

The present study explored stress and depression in adults ≥ 50 years of age. We also considered the possibility that certain chronic medical conditions may contribute to accelerated/accentuated aging [1,2,3,4], such that middle-aged individuals with comorbidities may be at increased risk for declines in physical, cognitive, and mental health compared to normatively aging adults. One such condition is chronic widespread pain (CWP), which affects at least 10–12% of adults. Examples of CWP include osteoarthritis, rheumatoid arthritis, low back and/or neck pain, complex regional pain syndrome, and fibromyalgia.

Fibromyalgia (FM) is a CWP characterized by muscle tenderness, joint pain, stiffness, and fatigue. Pain-related FM symptoms are often linked to physical and mental distress (e.g., abdominal pain, depression, anxiety, memory loss) [5,6]. The prevalence of FM is difficult to assess; diagnoses tend to be underestimated for men and overestimated for women, and there is still uncertainty in the accuracy of diagnoses [7,8]. However, there is extensive research on stress and how it affects pain, leading to increased risk of comorbid conditions and symptom severity [9,10,11]. The association of stress with FM and its role in the exacerbation of symptom severity remains complex.

Essentially, stressors precipitate stress, and depending on the context and coping resources available to the individual, stress may turn into distress (behavioral/mood responses [e.g., depression] resulting from stress) [12,13]. In the present study, stress was evaluated based on perceived stress using the Perceived Stress Scale (PSS) [14] and life stressors (life events, experiences, or changes that may lead to strain, or psychological, physiological, and behavioral outcomes) [15,16] using the Social Readjustment Rating Scale (SRRS) [17].

### 1.1. Perceived Stress

While the severity of stress varies among individuals, prolonged exposure can affect one’s ability to control and cope with perceived stress, especially with age. A cross-sectional study with a sample of participants with FM from different age groups (30–39, 40–49, 50–59, and 60–90 years) revealed that levels of perceived stress were higher for the oldest compared to the youngest age groups [18]. Chronic pain symptoms associated with FM may amplify stress, resulting in a cycle where individuals find themselves experiencing increased comorbid conditions associated with FM. The relationship between chronic pain and perceived stress may be bidirectional as well, such that stress may be exacerbated by or result from the symptoms of FM [19]. 

Perceived stress is also believed to be the driving mechanism behind many physical and mental health conditions, especially depression, which is commonly comorbid with FM [20,21]. Because perceived stress severity can depend on these comorbid conditions, the relationship between them can be difficult to assess. Significant associations have been found in common symptoms of FM, such as pain and stress [19]. Similar results between pain intensity and depression have been found in those with FM, who typically report higher levels of psychological distress [22]. Furthermore, the higher the severity of depressive symptoms, the greater the negative impact these symptoms have on pain severity, perceived stress, and quality of life compared to those without FM [22]. These results have also been found where those with FM had reduced functionality, greater perception of stress, and more severe depressive symptoms than those without chronic pain. Greater pain severity and decreased quality of life may also have a direct relationship with depressive symptoms in FM participants, suggesting that the comorbidities associated with depression are amplified in those with FM [23].

### 1.2. Life Stressors

While some life events can be positive and promote growth, recent adverse life events were found to be significantly linked to a higher risk of the onset of pain in a longitudinal study. The most common adverse life events predicting the onset of chronic pain included severe financial problems, separation from a partner, and/or the death of a family member or friend [24]. Based on this, it is important to observe how different types of stressors affect symptom severity. 

Past research also suggests that those with chronic pain may experience life stressors not found in healthier individuals. For example, Ghosh and Sharma [25] reported that those with chronic pain listed life events relating to changes in eating and sleeping habits as significant stressors. Other studies report more stressful issues related to employment for those with chronic pain [26]. Another study found that life stressors vary and impact health differently depending on factors such as race/ethnicity and/or socioeconomic status [27].

### 1.3. Distress/Depression

Both FM and depression share many similarities in pathophysiology that may lead to increased risk of and vulnerability to physical and environmental factors [28,29,30]. High co-occurrence of FM and depression has been found, with a bidirectional association between the two. Individuals with FM, despite no premorbid history of depression, may have an increased risk of depression. Similarly, those with depression and no prior history of FM may have an increased risk for chronic pain conditions [31]. Stress-related disorders affect physical, mental, and emotional health; however, symptoms may especially result from prolonged stress [13,32]. Moreover, recurring stressful experiences and depressive symptoms can trigger more symptoms of FM. While it is unclear whether higher levels of stress from major life events lead to FM, evaluating different types of stress may help us better understand how depression and FM impact individuals with FM [29,33].

The purpose of the current study was to see how the effects of pain and depression (using the Beck Depression Inventory) may impact perceived stress and responses to life stressors. 

We hypothesized that perceived stress and life stressors would be correlated in people with and without FM. We also predicted that individuals with FM, because of their chronic pain, would subjectively rate their perceived stress (measured by PSS) as more severe compared to controls. Persons with FM and/or depression were hypothesized to rate life stressors more severely than controls. We also predicted an interaction between FM status and depression, where individuals with FM and more depressive symptoms would report higher rates of stress severity compared to those without FM or depression.

## 2. Materials and Methods

The cross-sectional data used in the current study came from one wave of a larger longitudinal study conducted at the Fibromyalgia & Chronic Pain Center [1,34].

### 2.1. Participants

Seventy-three females and twenty-one males aged 50 to 93 years participated in this study; 53% had FM (see Table 1 for details). Potential participants had to be at least 50 years old and pass initial screening via telephone. Exclusion criteria and measures not included in the present study are described elsewhere (blinded for review). This study received Institutional Review Board (IRB) approval and participants provided informed consent. 

### 2.2. Measures

Basic demographic information such as age, gender, education, ethnicity, and income was collected [35]. In addition, other inventories were administered to assess FM symptoms, the severity of depressive symptoms, perceived stress, and life stressors. 

### 2.3. National Fibromyalgia Association Questionnaire (NFAQ)

The NFAQ was used to measure the impact of FM symptoms on basic physical function levels and general welfare. Derived from Bennett and colleagues [35], the NFAQ contains 23 items, including background information (age, sex, height, and weight) and questions regarding the extent to which participants experienced common FM symptoms within the past week. Participants were asked to rate their average level of functional abilities and psychological well-being on a scale from 0 to 10 (e.g., 0 = no pain to 10 = worst possible pain). Those with FM needed a physician’s diagnosis, and FM status was coded (0 = no; 1 = yes).

### 2.4. Beck Depression Inventory (BDI-II)

The BDI is a 21-item self-report inventory by Beck and colleagues for measuring depressive symptom severity. A revised version known as BDI-II [36] has been widely used as an assessment of the severity of depression. The questionnaire consists of 21 groups of statements where the participant must choose one statement in each group that best describes how the participant has felt during the past two weeks. Topics for these groups include loss of interest, worthlessness, and changes in sleeping patterns. BDI-II has been widely used as an indicator of depressive symptom severity but is not a diagnostic tool. Recent studies provide test–retest reliability ranging from 0.73 to 0.96 [37].

### 2.5. Cohen’s Perceived Stress Scale (PSS)

Perceived stress was measured using the PSS, which also measured participants’ current stress levels and which situations in their lives were appraised as stressful. The PSS is a 10-item questionnaire where individuals are asked about their feelings in the past month; it is scored on a 5-point scale ranging from 0 (Never) to 4 (Very often). Four PSS responses are then reverse-scored, and a sum is calculated to obtain a total, where higher scores suggest higher perceived stress [14]. The scale validity is strong (*r* = 0.72), as is the test–retest reliability (*r* = 0.93, *p* < 0.001) [38].

### 2.6. Social Readjustment Rating Scale (SRRS)

The SRRS is a 43-item questionnaire developed by Holmes and Rahe [17] identifying common major life stressors where the participant self-reports how severely that event has affected their lives. Each item is assigned a life change unit based on the severity of the life event (e.g., death of a spouse/significant other = 100; divorce = 73; marital separation = 65). Traditional coding for the SRRS inventory suggests that higher scores indicate greater stress severity. The SRRS yields high rank-order reliability and long-term stability ranging from *r* = 0.89 to 0.96 in healthy adults and *r* = 0.70 to 0.91 in psychiatric patients [39].

In our study, participants self-reported their own estimations of stressor severity by rating each real-life stressor experienced in the past year on a scale from 1 to 100 (SRRS: Participant). For stressors only, the difference between participants’ ratings and the SRRS standardized values was also calculated (SRRS: Difference) (i.e., [M Participant Rating Score—Standardized Score]). Higher scores indicated that self-reported severity exceeded standardized values, suggesting that participants found the stressors to be more severe than average.

### 2.7. Procedure

Participants were recruited by phone or email from the [university blinded] Fibromyalgia & Chronic Pain Center. Flyers were also distributed to local communities and/or via email. After initial eligibility screening, participants were mailed paperwork that included a consent form, basic demographic information (e.g., age, gender, education), past medical history, and self-report assessments including the PSS and SRRS. Participants brought completed forms to the site to assess performance measures in person. The BDI-II was also administered on site.

## 3. Results

Ninety-four participants (53% FM, 78% female) aged 50 to 93 years (*M* = 67.72, *SD* = 9.26) were included. Two participants were excluded from analysis due to incomplete BDI-II data. Demographics, select symptoms, and measures by FM status are presented in Table 1. 

Perceived stress was significantly different for FM versus non-FM participants (*t*(89) = −6.41, *p* < 0.001), such that individuals with FM reported more stress. For SRRS participant ratings, FM participants trended higher (*t*(92) = −1.78, *p* = 0.078). In addition, the SRRS difference (participant ratings minus standardized scores) was significant (*t*(92) = −4.21, *p* < 0.001). As shown in Table 1, FM participants on average exceeded SRRS standardized scores by 78 points, while the non-FM control group showed basically no difference (*M* = 0.09).

Of the 43 categories for the SRRS, 24 were reported by both FM and non-FM participants over the past year. Of these, five were significantly different for FM versus non-FM individuals, with ratings higher (more stressful) for those with FM (see Table 2). These included major changes in eating habits, major changes in sleeping habits, the death of a close friend, troubles with your boss, and major changes in working hours or conditions. In fact, except for the death of a close family member and major changes in the usual type and/or amount of recreation, the rest of the categories were higher for FM participants regardless of significance. Standardized scores for the five life events in Table 2 were 15, 16, 37, 23, and 20, respectively, indicating large increases in stress for those with FM.

The Pearson correlation analysis showed a significant correlation between PSS scores and SRRS participant ratings (*r*(94) = 0.33, *p* = 0.001), where such coding entailed higher ratings for more stressful events. The moderate positive correlation indicated that greater PSS scores were associated with higher SRRS scores. For the participant ratings minus the standardized scores for the SRRS, there was an even stronger positive correlation with PSS (*r*(94) = 0.45, *p* < 0.001). Table 3 shows these correlations along with those for potential covariates (age, pain, income, and depression). 

Three four-step hierarchical regression analyses were conducted to see how FM status and depression impacted subjective ratings of stress (PSS; SRRS: Participant; SRRS: Difference), after controlling for age and gender. Pain was not used in these regressions due to its association with FM status. In all regressions, predictors were step 1—age and gender, step 2—FM status, step 3—depression, and step 4—FM–depression interaction. With the addition of the interaction term, the depression variable was centered to reduce multicollinearity. Variables were screened for outliers, and analyses with outliers removed showed the same pattern of results as the entire sample. Results using the entire sample are therefore reported below.

### 3.1. Perceived Stress (PSS)

**Figure 1:**
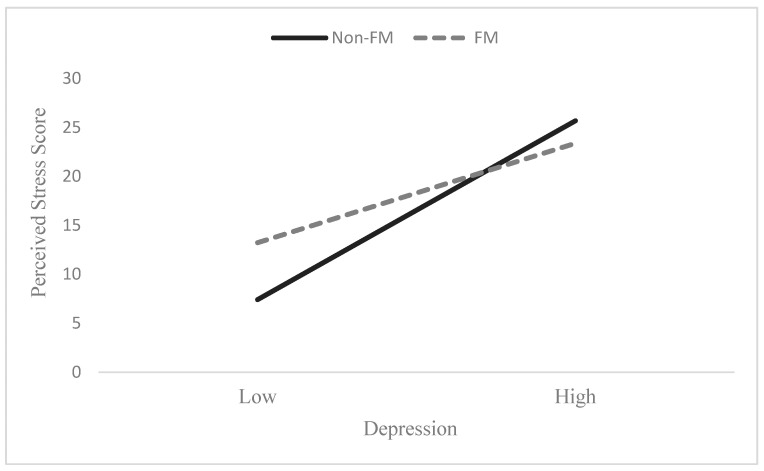
Interaction effect of FM status based on depression on perceived stress. Note: Depression categories were determined based on distribution of depression scores across sample (+/- 1 SD).

The main effects for both FM status and depression were significant in this model, where both those with FM and those with higher levels of depression (*F*(5, 88) = 28.14, *p* < 0.001) had greater perceived stress. Controlling for age and gender, the FM–depression interaction predicting perceived stress severity was also significant (*p* = 0.04). As illustrated in Figure 1, those with FM reported higher perceived stress when depression was low, but at high levels of depression, individuals without FM reported higher perceived stress. The results support the hypothesis that those with FM and/or depression would report higher ratings of perceived stress severity than their counterparts (see Table 4). 

### 3.2. Life Event Stressors (SRRS)

When controlling for age and gender, the FM–depression interaction predicting life stress severity was not significant for either ‘SRRS: Participant’ or ‘SRRS: Difference’. Thus, to conserve power, analyses reverted to the three-step model (step 1—age and gender, step 2—FM status, step 3—depression) for interpretation. 

With ‘SRRS: Participant’ as the criterion variable, the overall model was significant (*F*(4, 87) = 5.15, *p* < 0.001). Only depression was significant in the final model (*p* = 0.003).

With ‘SRRS: Difference’ as the criterion variable, the overall model was again significant (*F*(4, 87) = 13.72, *p* < 0.001). Older adults (*p* = 0.04) and men (*p* = 0.03) were less likely to report exaggerated stressor severity. In step 2, before depression was added to the model, individuals with FM were significantly more likely to report stressor ratings above standardized scores in the SRRS measure (*p* = 0.01). However, when depression was added, FM status was no longer significant (*p* > 0.05). These results support the hypothesis that those with FM and/or depression would report higher life stressor severity, but the impact was greater for those with depression regardless of FM status, as it became the driving factor when all variables were entered into the model (*p* = 0.002); see Table 4. 

## 4. Discussion

In our study, significant associations were found between perceived stress and life stressors; however, important distinctions were also evident. The results support the hypothesis that those with FM were significantly more likely to report higher levels of stress above standardized scores on both the PSS and SRRS. Additionally, those with FM and depression reported the experience of perceived stress and life stressors as more severe than those without FM, supporting our second hypothesis. Interestingly, depressive symptoms played a more significant role than FM status in predicting life stressors. Although a significant interaction between FM status and depression was found only for perceived stress (PSS), because our sample and much of the FM population are more likely to be depressed, depressive symptoms in FM seem to be driving the relationship for both stress measures. When controlling for depressive symptoms predicting SRRS, FM status was no longer significant, suggesting that distress is a more important factor in predicting life stressor ratings. The evaluation of the relationship between depression and FM should continue to be explored as FM status is commonly comorbid with depression.

In addition, those without FM who had lower levels of depression scored lower for perceived stress, whereas the non-FM group with higher levels of depression scored notably higher on the PSS than those with FM and high depression, only partially supporting our third hypothesis (see Figure 1). One possible explanation may be that those with chronic pain and depression actively seek more alternative methods of stress management (e.g., practicing mindfulness, pain management techniques, lifestyle changes) for both conditions, so their stress levels may be more controlled [40]. 

The findings also emphasize how different types of stressors can impact stress severity. Our FM group only endorsed slightly more life stressors on the SRRS (35 vs. 31 events). They were, however, more likely to rate life stressors as more severe than non-FM participants (SRRS: Participant) and significantly rated particular events as more stressful than standardized scores (SRRS: Difference), with even mild stressful events rated as more severe [41]. Despite small numbers, certain environmental factors were particularly stressful for individuals with FM in our study (changes in eating habits, declines in sleep quality, the death of a close friend, employment issues), consistent with prior research in individuals with chronic pain. For example, diet and eating behaviors were reported as stressful events for FM individuals in our study, in concert with [25,32]. Seib and colleagues [32] also found that sleep disturbances were related to increased stress, more depression, and lower quality of life in those with chronic pain; see also [25]. 

Regarding the death of a close friend, the few studies on grief and chronic pain suggest that these factors influence each other bidirectionally and appear to share certain underlying neuroanatomical mechanisms [42,43]. Employment issues while working with chronic pain provided more life stress for those with FM compared to healthy controls in terms of trouble with bosses and/or major changes in working hours/conditions. This has also been reported elsewhere [25]. Moreover, Palstam and Mannerkorpi [44] conducted a systematic review of both quantitative and qualitative studies on employment in those with FM and found that certain working conditions contributed to higher rates of disability in this group. Added stress also occurred in those trying to hide their condition from employers. Those who had support regarding their condition from management as well as colleagues fared better. In summary, it has been suggested that those with chronic pain demonstrate clusters of life stressors consisting of “bereavement, financial, familial, and self- and job-related stressors” [25].

Managing stress may help with additional factors that negatively impact chronic pain. Current stress management strategies include, but are not limited to, practicing relaxation techniques (e.g., yoga, meditation, practicing mindfulness), exercising regularly, and staying well rested. Mindfulness interventions in those with FM or chronic pain have also been shown to be effective in alleviating depressive symptoms and pain interference while improving psychological health and quality of life [45,46,47,48]. Studies with older adults diagnosed with depression also suggest that increased social interaction and social support may be associated with fewer or less severe depressive symptoms [49].

## 5. Limitations

Much of our sample was female; thus, recruiting more male participants and increasing the sample size of those with and without FM may improve generalization. Self-report measures regarding perceived stress and life stressors may not have accurately reflected cognitive appraisal related to current emotions or life events. Additionally, most of our sample had mild to moderate depression, with very few having severe levels of depression; more significant findings may be found with a greater distribution of those scoring higher on the depression inventory. Furthermore, most of our sample with FM tended to be relatively high-functioning. As the study design was cross-sectional, no inferences could be made regarding cause and effect. Future research should consider longitudinal designs to address this limitation.

## 6. Future Research 

The inclusion of anxiety may offer additional information on how different chronic conditions might exacerbate FM and depressive symptoms. Life stressors also play a role in distress and are commonly associated with anxiety disorders, suggesting that such occurrences can act as precipitating factors [29]. 

Future research might also examine socioeconomic status (SES). Specifically, life circumstances such as education, income, living conditions [27], and age [50] may further help researchers understand the intersection of sociodemographic factors and stress. Moreover, exploring income highlights issues related to less access to resources for pain management, which can further contribute to symptom severity due to economic and racial/ethnic disparities [51,52]. 

A much broader range of psychosocial variables should be considered in future research as well. One study suggests that factors such as childhood neglect, sexual assault, lower income and education levels, and increased body mass index may contribute to increased likelihood of FM [28]. Another study suggests that symptoms of chronic pain and depression may be exacerbated by lower levels of health literacy [53]. Finally, Quinn et al. [4] propose that health-related outcomes may be related to affect balance (the difference between positive and negative affect) with this balance associated with the PASTOR theory of resilience [54]. This suggests that interventions which focus on positive affect may provide for increased resiliency, which might then protect against more negative health outcomes.

Overall, our study suggests the need for a stronger focus on the health of middle-aged and older adults with comorbidities, as well as understanding different types of stress and their impact on individuals suffering from other health issues such as CWP. This study’s findings supplement other research addressing the vulnerability of FM and other chronic pain groups whose symptoms are exacerbated by depression and participant perceptions of stress. While we cannot prevent stressful situations or life stressors, reducing other factors related to stress and improving access to psychological health interventions can enhance strategies for stress management.

## Figures and Tables

**Table 1:** Demographics, symptoms, and stress measures by FM status.

Variables	FM (*n* = 50)		Non-FM (*n* = 44)		
	M/%/Med	SD/Range	M/%/Med	SD	*p*
Gender (% female)	88		66		0.01
Age	63.62	7.37	72.40	9.04	<0.001
Pain	5.22	2.22	2.01	1.95	<0.001
Fatigue	6.40	2.39	2.64	1.95	<0.001
Anxiety	4.47	3.16	1.30	1.90	<0.001
Income (SES)	$65 K	<$9 K to >$200 K	$85 K	<$9 K to >$200 K	0.314
BDI-II score ^a^	15.48	10.25	4.80	5.18	0.001
PSS score ^b^	21.30	8.08	10.78	7.42	0.001
SRRS: Participant ^c^	89.16	86.36	60.07	69.81	0.078
SRRS: Difference ^d^	78.16	114.23	0.09	48.52	<0.001

Note: Pain, fatigue, and anxiety variables rated on a scale of 0 to 10 from NFA questionnaire. ^a^ Beck Depression Inventory-II score. ^b^ Perceived Stress Scale score. ^c^ Social Readjustment Rating Scale: Participant ratings. ^d^ Social Readjustment Rating Scale: Participant ratings—standardized scores; higher scores indicate more severe stressor ratings.

**Table 2:** Life stressors from SRRS ^a^ based on FM ^b^ status.

Life Event		FM ^b^ (*n* = 50)	Non-FM (*n* = 44)		
	N	M (SD)	N	M (SD)	*p*
Major change in eating habits (a lot more or less food intake, or very different meal hours or surroundings)	11	55 (26.17)	2	10 (0.00)	0.04 *
Major change in sleeping habits (a lot more or a lot less than usual)	5	65 (23.98)	4	26.25 (17.97)	0.03 *
Death of a close friend	3	60 (10.00)	5	31 (11.40)	0.01 *
Troubles with your boss	4	75 (10.80)	1	20 (0.00)	0.02 *
Major changes in working hours or conditions	3	65 (13.23)	2	20 (14.14)	0.04 *

Note. ^a^ SRRS = Social Readjustment Rating Scale. ^b^ FM = fibromyalgia. * *p* < 0.05.

**Table 3:** Correlation matrix for study variables and potential covariates.

Variables	1	2	3	4	5	6	7
1. Age	–						
2. Pain	−0.37 **	–					
3. Income	−0.04	−0.22 *	–				
4. BDI-II Inventory	−0.40 **	0.60 **	−0.26 *	–			
5. PSS	−0.42 **	0.59 **	−0.24 **	0.76 **	–		
6. SRRS: Participant	−0.31 **	0.19	−0.12	0.38	0.33	–	
7. SRRS: Difference	−0.38 **	−0.26 *	−0.21 *	0.52 **	0.45 **	0.43 **	–

Note: BDI-II = Beck Depression Inventory-II, PSS = Perceived Stress Scale, SRRS = Social Readjustment Rating Scale, * *p* < 0.05, and ** *p* < 0.01.

**Table 4:** Hierarchical regression analyses predicting perceived stress with PSS (4-step model) and major life stressors with SRRS (3-step model).

Variables	Age	Gender	FM Status	BDI_II	FM X BDI ^a^	ΔR^2^
Perceived Stress Scale						
B	−0.11	0.17	1.88	0.93	−0.41	0.02 *
SE B	0.08	1.63	1.75	0.18	0.20	
β	−0.11	−0.01	0.10	0.97 ***	−0.35 *	
SRRS: Participant						
B	−1.66	23.86	−23.27	2.90		0.09 **
SE B	0.95	18.82	19.50	0.95		
β	−0.19	0.13	−0.15	0.36 **		
SRRS: Difference						
B	2.03	−42.90	−25.31	−3.21		0.08 **
SE B	0.98	19.47	20.18	0.99		
β	0.20 *	−0.19 *	−0.14	−0.33 **		

Note: Coefficients are from the final 4-step model for PSS (step 1—age, gender; step 2—FM or non-FM; step 3—depression; step 4—FM X depr) and 3-step model for SRRS (step 1—age, gender; step 2—FM or non-FM; step 3—depression). ^a^ FM–depression interaction. * *p* < 0.05, ** *p* < 0.01, and *** *p* < 0.001.

## Data Availability

Data are contained within this article.

## Abbreviations

BDI-II	Beck Depression Inventory
CWP	chronic widespread pain
FM	fibromyalgia
NFAQ	National Fibromyalgia Association Questionnaire
Non-FM	no fibromyalgia; healthy control participants
PASTOR	Positive Appraisal Style Theory of Resilience
PSS	Perceived Stress Scale
SRRS	Social Readjustment Rating Scale
SRRS: Difference	Social Readjustment Rating Scale: Participants’ own rating of real-life stressors compared to standardized scores (1 to 100)
SRRS: Participant	Social Readjustment Rating Scale: Participants’ own rating of real-life stressors in past year (1 to 100)
